# Tool changing and tool sharing system for interconnected multi-material direct ink write 3D printers

**DOI:** 10.1016/j.ohx.2025.e00681

**Published:** 2025-07-28

**Authors:** James P. Verheyden, Bryce Huffaker, Max J. Sevcik, Isaac Snyder, Finnegan Wilson, Grace I. Rabinowitz, Carter Watkins, Elbert Caravaca, Edward G. Tersine, Veronica Eliasson

**Affiliations:** a Colorado School of Mines Department of Mechanical Engineering, Metallurgical and Materials Engineering, Computer Science, Colorado School of Mines, Golden, CO 80401, United States of America; b Picatinny Arsenal, US Army Combat Capabilities Development Command - Armaments Center (DEVCOM-AC), Picatinny, NJ 07806, United States of America; c Naval Surface Warfare Center Indian Head Division, Indian Head, MD 20640, United States of America

**Keywords:** Additive manufacturing, Direct ink write, Tool changing

## Abstract

Direct ink write (DIW) is a material extrusion additive manufacturing technique where a flowable liquid or semi-solid ink is selectively deposited through a nozzle onto a build surface. DIW can be leveraged to print a wide range of multi-material components with unique geometries, which would otherwise be challenging to produce with traditional manufacturing techniques. However, despite their unique capabilities, DIW extruders are costly to purchase and labor intensive to clean, limiting widespread adoption. To address these challenges, an automatic tool changer with tool sharing system was developed to reduce printer capital costs, minimize operator interactions, and increase extruder utilization. Unlike conventional tool changers popular with fused filament fabrication (FFF) printers, which allows a single printer to swap between multiple tools, this novel tool sharing system enables tool sharing and coordination between two adjacent printers. By effectively halving the total number of extruders and associated cleaning operations, DIW capabilities are greatly improved, increasing production, lowering system cost, and minimizing operator involvement. The hardware consists of a tool changing mechanism, post processor to insert tool exchange G-code, and software to coordinate tool sharing between printers, enabling tool changing and tool sharing between two adjacent printers.

## Graphical abstract

**Figure:**
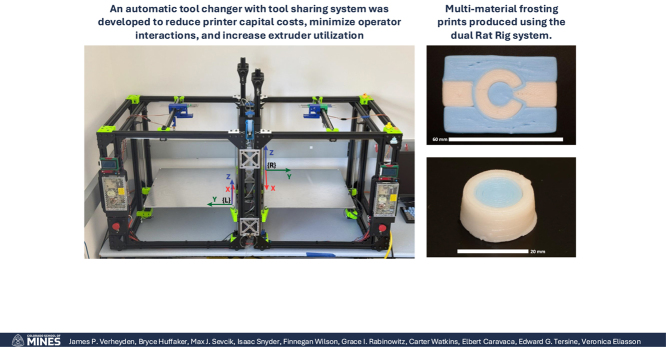

## Specifications table

**Table:** 

Hardware name	Dual Printer Automatic Tool Changer for DIW 3D Printing
Subject area	Engineering and Materials Science
Hardware type	Mechanical Engineering and Materials Science
Closest commercial analog	Rat Rig V-Core 3.1 + E3D ToolChanger
Open source license	TAPR OHL
Cost of hardware	$14,200
Source file repository	http://doi.org/10.17632/kypz3jnntb.1

## 1 Hardware in context

Direct ink write (DIW) is an additive manufacturing technique where a flowable liquid or semi-solid ink is selectively deposited onto a build by extruding the material through a nozzle [1]. Unlike fused filament fabrication (FFF), another material extrusion process, where a thermoplastic is extruded through a hot end, DIW does not rely on heating to dispense material, enabling a wide range of paste-like feedstocks to be printed [2]. DIW has a multitude of applications with a wide range of inks including polymers [3], [4], ceramics [5], [6], composites [7], and biological materials [8], [9]. While previous work has shown that muli-material and even compositionally graded prints can be achieved with DIW [10], [11], adding extruders to a printer gantry increases printer head weight and decreases functional print volume. This hardware seeks to improve DIW multi-material printing by developing an automatic tool changer that can share tools between two adjacent printers.

Unlike FFF hot end extruders which frequently need minimal to no cleaning between uses, DIW extruders often require extensive cleaning operations after each use. Continuously operated DIW extruders, such as those used in assembly lines may not require daily cleaning, so long as material is not left to cure in the extruder. In applications requiring intermittent extrusion, such as those advantageous to rapid manufacturing, DIW extruders must be cleaned before periods of inactivity to prevent ink from hardening and causing internal damage. Reducing extruder cleaning and overall operator exposure is even more important when working with hazardous inks. Considering maintenance and cleaning operations are necessary when developing a DIW printing system. By developing a tool changer system which can share tools between two printers, the total number of extruders can be decreased. Considering most printing applications, especially those involving multiple materials, only use one extruder at a time, sharing tools can double extruder utilization. Tool sharing allows tools not currently used by one printer to be used by the other printer. This greatly improves multi-material 3D printing capabilities by enabling two printers to produce multi-material parts while sharing just two extruders. Tool sharing also reduces ink contacting components and enables fewer larger ink reservoirs to be used, reducing waste from leftover residue ink. Additionally, tool sharing reduces capital costs by decreasing the number of required extruders, which can comprise a significant proportion of the total printer cost [11].

Several tool changer designs for 3D printers exist [8], [12], [13], [14], [15], many of which require minimal alteration to accommodate DIW hardware. However, incorporating tool sharing and enabling tool exchanges from two adjacent printers necessitates the development of a custom tool changer.

To facilitate tool changing between two adjacent printers, a suitable printer design was selected which could not only accommodate a tool changer, but also move the tool to a mutually accessible area for tool sharing. From these limitations, a prospective printer design should allow for translation of the extruder in at least one, but preferably two directions. This criteria eliminates printer designs in which the extruder is stationary, and the print bed moves in three orthogonal directions. Out of the several printer designs which have the extruder move with respect to the printer frame, a core XY design was selected. Core XY is a parallel belt driven design which achieves cartesian planar motion while minimizing gantry weight by locating the two driving stepper motors on the printer frame [16]. Two belts connected to two separate stepper motors work in tandem to translate the tool head along a gantry in the X-direction and translate the entire gantry in the perpendicular Y-direction. A tool changer can be incorporated with a core XY printer by locating the tool docks along the X-direction. For tool sharing, the docks should be located on a shared side which is reachable by both printers. While the task space of each printer only covers the build plate or portion thereof, to share tools, the workspace, or reachability, of each printer must overlap [17].

Previous work has shown that ViscoTec eco-PENs can be used to selectively deposit visco-elastic inks and can be modified to print on a Prusa using a NEMA 17 stepper motor and custom 3D printed adapters [18]. ViscoTec eco-PENs work by using a progressive cavity pump actuated by a stepper motor to precisely dispense a viscous paste with microliter accuracy [19]. These extruders can be modified to be compatible with the native functionality of many 3D printer firmwares by removing the original servo motor and replacing it with a conventional 3D printer stepper motor using a custom 3D printed adapter [11], [18]. Modifying the adapter into a tool holder can enable the extruder to be grabbed by the tool mount and returned to the dock. While each eco-PEN300 extruder costs around $4500, incorporating tool sharing reduces overall printer system cost by increasing extruder utilization. Additionally, integrating an eco-PEN onto a consumer 3D printer remains much more cost effective than buying a commercial DIW printer such as the F-NIS made by Sygnis or building an enterprise-grade printer such as that described by Kline et al. [10].

**Fig. 1:**
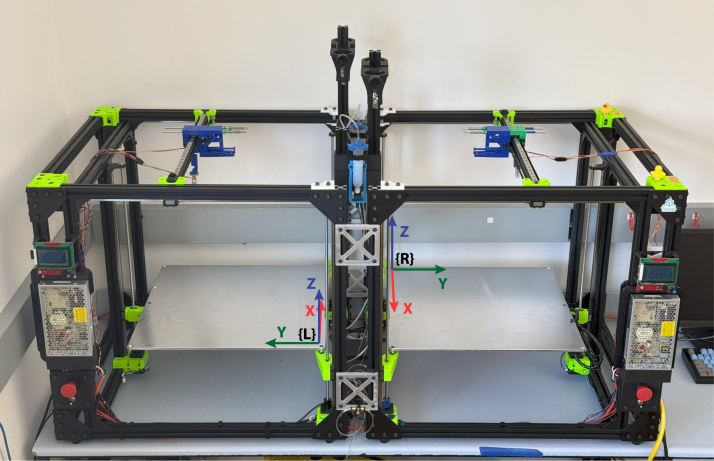
Two interconnected modified Rat Rig V-Core 3.1 printers. Note the reference frames for the left ({L}) and right ({R}) printers and how frame {R} is rotated 180° about the Z axis with respect to {L}.

## 2 Hardware description

The dual printer automatic tool changing system consists of two conjoined printers with a shared tool dock allowing for either printer to tool change between shared tools (Fig. 1). Motion for each printer is separately controlled with the stock BIGTREETECH (BTT) Octopus Pro v1.1 mainboard included with the Rat Rig V-Core 3.1 printer. An Arduino Mega extruder controller allows for coordinated dispensing of the extruders between the two printers. Custom Python and C++ software was developed to supervise changing operations, actuate dispensers, and coordinate printing operations.

### 2.1 Printers

Two commercially available Rat Rig V-Core 3.1 printers were selected for modification. Rat Rig was chosen due to its open-source design, large size, and ease of customizability. The Rat Rig V-Core 3.1 utilizes a core XY gantry system and has a 500 mm × 500 mm × 500 mm build volume providing sufficient space along the X-direction to install two tool changer docks. The core XY design is compatible with installing tool changers as it minimizes weight by locating motors on the stationary frame and not the mobile gantry. The tool head can translate in the X-direction along the gantry and the gantry can translate in the Y-direction. Therefore, tool docks are located along the X-direction, allowing the tool mount to select between different tools by translating along the gantry (Fig. 1).

The printers were modified by removing bed heating functionality which is not needed for DIW printing and rearranging supporting aluminum extrusion to accommodate inter-printer tool sharing. The upper support extrusion was lowered to allow for tool holders to be passed across to the adjacent printer. Conventional 3D printer tool changers, such as the E3D and Jubilee motion system, dock on one side of a tool and pick up on the opposite side, allowing for space efficient storage of tools by only requiring movement in the Y-direction [8], [15]. This configuration does not allow for an adjacent printer to pick up the tool. Instead, the tool holders were designed to allow for picking up on either end, with docking attachments on the side. This allows the tool to be picked up and docked by either printer.

Connecting the two printers together involved rotating one of the printers 180 degrees and securing both printers together with custom machined brackets. Power switches and emergency stop buttons that disable motor function were independently wired for each printer. Additionally, for added safety, a single emergency stop button with two separate normally-closed switches was installed to disconnect motor power from both printers, while isolating each printer’s power circuits.

Each printer was flashed with an identical copy of modified Marlin (2.0.x) firmware. The open-source firmware Marlin was selected due to its customizability and ability to operate without wireless data transmission.

**Fig. 2:**
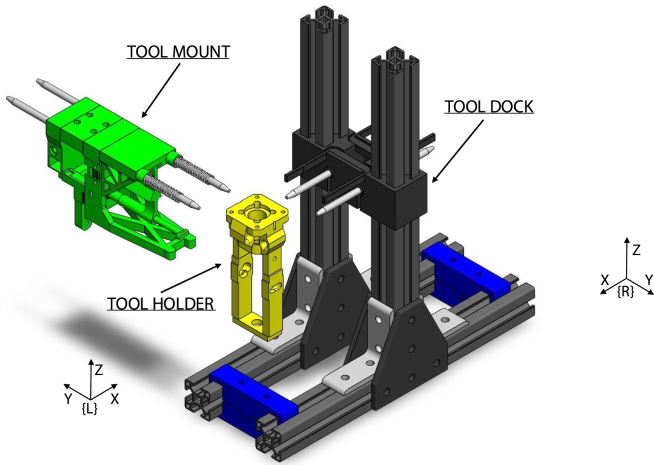
Dual printer tool changer system with labeled tool mount, tool holder, and tool dock components. Includes commercial off the shelf (COTS) components [20].

**Fig. 3:**
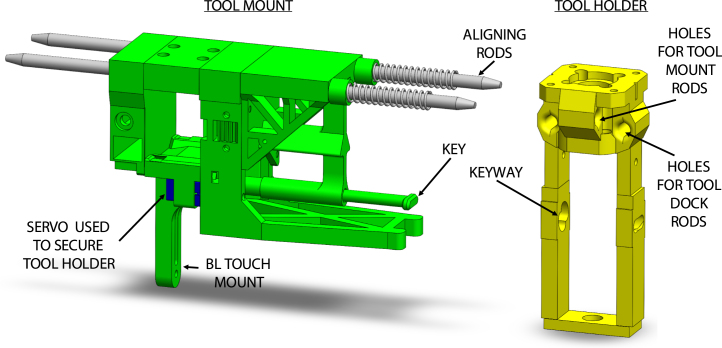
Tool mount and tool holder design features. Includes COTS spring [20].

### 2.2 Tool mount

**Fig. 4:**
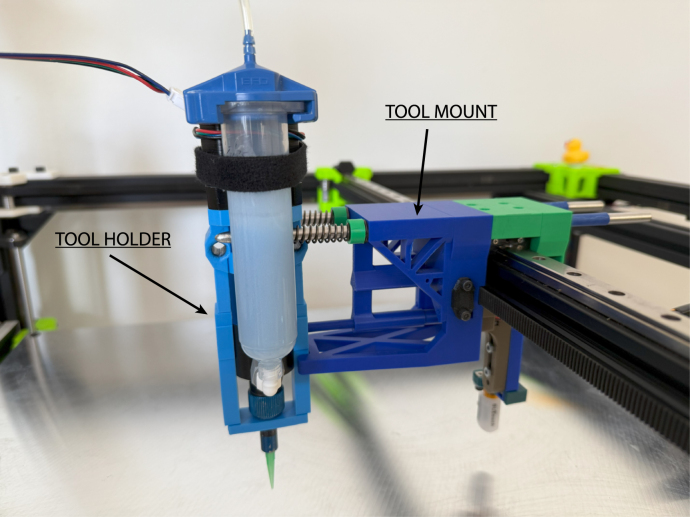
Tool mount grasping a tool holder.

Tools, held by a tool holder, are picked up by the tool mount with two rounded stainless steel dowels (Fig. 2). The tool holder is secured via a keyway system actuated by a servo motor. Springs on the dowels prevent vibration by applying a constraining force to the tool holder. The tool mount is attached to the printer’s gantry and also includes attachment for a Z-homing BLTouch. To pick up a tool, the tool mount connects to the tool holder by translating in the Y-direction, inserting the dowels, and securing with the servo actuated key and keyway. To remove the tool holder from the dock, the tool mount translates in the X-direction to remove the tool holder from the dock. Replacing the tool follows a similar procedure in reverse. An annotated model of the tool mount is shown in Fig. 3 and a picture of the tool mount holding a tool holder is shown in Fig. 4.

### 2.3 Tool holder

A custom 3D printed attachment was designed to hold a ViscoTec eco-PEN300 and interface with the tool changer (Fig. 2). A 3D printed adapter design by Sevcik et, al. that allows the eco-PEN to be controlled by a stepper motor was further modified for tool changing [18]. The attachment was designed to permit easy removal of the eco-PEN for cleaning and maintenance operations. Holes on the tool holder interface with dowels on the tool mount and tool dock to permit pick-up and return of the tool. The fixture was designed to allow the tool to be picked and placed on both sides by both printers from a single home position. This was accomplished by positioning the tool holder and dock dowel holes perpendicular to each other, separating motion associated with picking up and removing the tool from the dock. A keyway interfaces with a servo actuated key to secure the tool holder to the tool mount. An annotated model of the tool holder is shown in Fig. 3.

### 2.4 Tool dock

The tool dock was designed to fit in between the two printers and to allow tool changing by both printers’ tool mounts. Aligning rods are used to hold the tools in the home position and a clip is used to prevent tools from sliding off the rods due to vibration. Unlike conventional 3D printer tool docks where tool parking is achieved by motion in the Y-direction, this tool dock operates by moving the tools in the X-direction to park (Fig. 2). This allows the tools to be picked up by an adjacent printer.

### 2.5 Tool changing post processor

A custom post processor script was developed to facilitate tool changing. 3D models were sliced in Simplify3D due to its high degree of tool path and extrusion setting customizability. To accomplish tool changing, a code flag such as “;pickup_1” was inserted into the tool changing code insertion window in Simplify3D. While switching between two tools could be achieved with native Simplify3D functionality, increasing the number of tools above two introduced problems stemming from replacing the tool in the correct location before picking up another tool. To solve this problem, a custom post processor script was developed which inserts tool change G-code into the print file at locations of code flags while keeping track of which tool a printer currently has and where it must be returned to.

### 2.6 Printer coordination

An Arduino Mega 2560 REV3 microcontroller running a custom C++ script coordinates tool sharing between printers. Each printer retains its own mainboard, but uses four GPIO pins connected to the Aurduino to request and wait for extruder availability. When a printer encounters a pickup tool change flag in the G-code, it signals the Arduino with pin J45 and specifies the extruder (0–3) via binary with pins J48 and J49. Pins can be set to either high (5 V) or low (0 V) and follow standard binary convention. For example, if there are two pins where one is at a high state and the other at a low state, the Arduino will register this input as a request to pick up extruder 2, or in binary: 10. This allows the Arduino to handle requests for up to four extruders. More extruders can be handled by adding additional request pins, which will increase the number of extruders to 2n where n is the number of wires designated as a request signal.

During the extruder selection process, the printer waits for a response from the Arduino using G-code command “M226” which causes the printer to wait for pin J44 to reach a particular state before continuing. This pauses the printer until the next desired tool is available. The Arduino determines if the desired extruder is available to be picked up via a standard interrupt, where the Arduino checks the location of the extruder. If the extruder is at the tool dock, the Arduino sends the appropriate response signal. If the extruder is currently being used by another printer, the Arduino will wait until that extruder has been put down before sending the response. Once the response signal has been received, the printer will pick up the requested extruder. A SunFounder 8 channel relay controlled by pins on the Arduino switches which printer is connected to an extruder. Motor wires from the left and right printers are connected to the normally open and normally closed sides of the relay and the extruder is connected to the common output (see Fig. 10). To tool change, the Arduino specifies which printer a motor should connect to with high and low pin states. After tool changing, the printer will continue executing G-code until the conclusion of a print where the printer will return its tool to the tool dock.

### 2.7 Hardware overview summary

• An automatic tool changer is able to share tools between two adjacent 3D printers
• This printer has been modified to be compatible with DIW printing, allowing for selective deposition of viscous inks
• Two 3D printers are integrated together for increased throughput with reduced extruder investment and maintenance operations
• Multi-material printing capabilities are possible

## 3 Design files summary

Files included within the article are listed in Table 1. The tool mount, tool holder, and dock were 3D printed in PLA from the STL files. The custom software was used to facilitate tool changing and printer coordination. Additional hardware and materials used in the construction of the tool changers are described in the Bill of Materials.

**Table 1:** Files available with the article.

Design filename	File type	Open source license	Location of the file
Double Sleeve Mount - Snap Connector	.STL	TAPR OHL	Available with the article
Double Sleeve Mount - Spacer_1mm	.STL	TAPR OHL	Available with the article
Double Sleeve Mount - Spacer_5mm	.STL	TAPR OHL	Available with the article
Double Sleeve Mount - Spacer_30mm	.STL	TAPR OHL	Available with the article
Double Sleeve Mount - Spacer_85mm	.STL	TAPR OHL	Available with the article
Double Sleeve Mount - Upper	.STL	TAPR OHL	Available with the article
Rail Mount - Back	.STL	TAPR OHL	Available with the article
Rail Mount - BLT Mount	.STL	TAPR OHL	Available with the article
Rail Mount - Connector	.STL	TAPR OHL	Available with the article
Rail Mount - Front Support Plate	.STL	TAPR OHL	Available with the article
Rail Mount - Front	.STL	TAPR OHL	Available with the article
Rail Mount - Servo Rod Support	.STL	TAPR OHL	Available with the article
Rail Mount - Spring Holder	.STL	TAPR OHL	Available with the article
Servo Gear Shaft Coupling	.STL	TAPR OHL	Available with the article
Servo Lock Key	.STL	TAPR OHL	Available with the article
Tool Housing - Key Slot	.STL	TAPR OHL	Available with the article
Tool Housing - Nozzle Clip	.STL	TAPR OHL	Available with the article
Tool Housing - Stepper Motor Mount Adapter	.STL	TAPR OHL	Available with the article
BLtouch_arm	.STL	TAPR OHL	Available with the article
BLtouch_bracket	.STL	TAPR OHL	Available with the article
Rotor Adapter	.STL	TAPR OHL	Available with the article
RatRigSpacerPlate	.STL	TAPR OHL	Available with the article
xy_idle_lower_new	.STL	TAPR OHL	Available with the article
xy_idler_upper_new	.STL	TAPR OHL	Available with the article
Tool Dock Rod	.STL	TAPR OHL	Available with the article
Tool Mount Rod	.STL	TAPR OHL	Available with the article
Marlin-bugfix-2.0.9.3.x	.zip	TAPR OHL	Available with the article
ToolExchangerPostProcess	.py	TAPR OHL	Available with the article
ExtruderInterface	.zip	TAPR OHL	Available with the article
ToolExchangeGcode	.zip	TAPR OHL	Available with the article
S3D	.factory	TAPR OHL	Available with the article
Bill of Materials	.xlsx	TAPR OHL	Available with the article

**Table 2:** Required software.

Software	Link	Price
Pronterface	https://github.com/kliment/Printrun/releases/tag/printrun-2.0.0rc8	Free
Simplify3D	https://www.simplify3d.com/buy-now/	$199
Marlin	https://github.com/MarlinFirmware/Configurations/tree/bugfix-2.0.x	Free
Arduino IDE	https://www.arduino.cc/en/software	Free
Visual Studio Code	https://code.visualstudio.com/	Free

### 3.1 Brief description of files

• **Double Sleeve Mount – Snap Connector:** 3D printed plastic part used to secure the tool holder in the dock and prevent the tool holder from sliding off due to vibration. Interference fits onto *Double Sleeve Mount – Upper*. One required for each extruder.
• **Double Sleeve Mount – Spacer:** 3D printed plastic part used to raise and position the *Double Sleeve Mount – Upper*. Available in 1 mm, 5 mm, 30 mm, and 85 mm heights. Part height can be modified in CAD to achieve the desired height offset. The offset used in this design is approximately 86 mm. Clearance fits with 30 mm 80/20 aluminum extrusion. Two sets required for every two extruders. Note: when stacking spacers, height tolerances can compound.
• **Double Sleeve Mount – Upper:** 3D printed plastic part which interference fits with the *Tool Dock Rods* to hold the tool holders in the docked position. One required for every two extruders.
• **Rail Mount – Back:** 3D printed plastic part which connects the tool mount with the printer’s timing belt. Interference fits with the *Tool Mount Rods*. One required for each printer.
• **Rail Mount – BLT Mount:** 3D printed plastic part which provides an adjustable location for a BLTouch to be mounted using additional 3D printed parts. Interference fits with the adjacent printed components. One required for each printer.
• **Rail Mount – Connector:** 3D printed plastic part which connects the tool mount with the printer’s gantry linear rail. Interference fits with the *Tool Mount Rods*. One required for each printer.
• **Rail Mount – Front Support Plate:** 3D printed plastic part which holds the *Tool Mount Rods* with an interference fit. Connects to *Rail Mount – Front* and *Rail Mount – Servo Rod Support* with interference fits. One required for each printer.
• **Rail Mount – Front:** 3D printed plastic part which holds the *Tool Mount Rods* with an interference fit and prevents X-direction motion of the tool holder. Connects to *Rail Mount – Front Support Plate* with an interference fit. One required for each printer.
• **Rail Mount – Servo Rod Support:** 3D printed plastic part which secures the servo rod. Interference fits with *Rail Mount – Front Support Plate*. One required for each printer.
• **Rail Mount – Spring Holder:** 3D printed plastic part which holds the spring and prevents the spring from sliding along the *Tool Mount Rods*. Interference fits with rods. Two required for each printer.
• **Servo Gear Shaft Coupling:** 3D printed plastic part which connects the *Servo Lock Key* with the servo. Note: this part is glued onto the *Servo Lock Key*, but only after properly inserting the *Servo Lock Key* through the *Rail Mount – Servo Rod Support*. One required for each printer.
• **Servo Lock Key:** 3D printed plastic part which interfaces with the tool holder to secure the tool. Assembled by inserting through the *Servo Rod Support* before gluing to the *Servo Gear Shaft Coupling*. One required for each printer.
• **Tool Housing – Key Slot:** 3D printed plastic part on the tool holder which interfaces with the *Servo Lock Key*. Interference fits with the *Tool Housing – Nozzle Clip* and *Tool Housing – Stepper Motor Mount Adapter*. Two are required for each extruder.
• **Tool Housing – Nozzle Clip:** 3D printed plastic part on the tool holder which aligns the extrusion end of the eco-PEN. Interference fits with the *Tool Housing – Key Slot*. One required for each extruder.
• **Tool Housing – Stepper Motor Mount Adapter:** 3D printed plastic part on the tool holder which interfaces with the *Tool Dock Rods* and *Tool Mount Rods* with a clearance fit. Interference fits with *Tool Housing – Key Slot*. One required for each extruder.
• **BLtouch_arm:** 3D printed plastic part which connects to *BLtouch_bracket* and *Rail Mount – BLT Mount* and allows for vertical adjustment of the BL Touch sensor. One required for each printer.
• **BLtouch_bracket:** 3D printed plastic part which holds the BL Touch. One required for every printer.
• **Rotor Adapter:** 3D printed plastic part which couples a NEMA 17 stepper motor to the eco-PEN. One required for each extruder.
• **RatRigSpacerPlate:** Aluminum CNC machined bracket to connect the two Rat Rig printers. Four required for each two printers.
• **xy_idler_lower_new:** 3D printed plastic part which replaces the Rat Rig stock CoreXY idler plate. Two required for every printer.
• **xy_idler_upper_new:** 3D printed plastic part which replaces the Rat Rig stock 3D printed CoreXY idler. Two required for every printer.
• **Tool Dock Rod** Metal component fabricated by cutting to length and rounding the ends of a 6 mm diameter 304 stainless steel rod. Two required for every two extruders.
• **Tool Mount Rod** Metal component fabricated by cutting to length and rounding the ends of a 6 mm diameter 304 stainless steel rod. Two required for every printer.
• **Marlin-bugfix-2.0.9.3.x:** Modified Marlin bugfix-2.0.x configured for tool sharing and tool changing on the Rat Rig.
• **ToolExchangerPostProcess:** Python script to insert tool change G-code at code flags within Simplify3D sliced file. Update with any tool change G-code variations.
• **ExtruderInterface:** Code to facilitate and coordinate tool sharing operations between two printers.
• **ToolExchangeGcode:** Example tool change G-code. May need to be adjusted to align with tool dock towers. Insert tool change G-code into G-code post processor after setup. Translation F speed may be increased.
• **S3D:** Simplify3D factory file with configured extruder settings and example part.

## 4 Bill of materials summary

The Bill of Materials is included in the file repository associated with this article. The software required to implement the hardware is listed in Table 2.

## 5 Build instructions

### 5.1 Rat rig assembly

• Assemble the Rat Rig V-Core 3.1 per manufacturer’s instructions. Deviations from standard assembly procedures include:
  – Not installing the heated bed, SSR relay, thermistors, or fans.
  – Not installing the Raspberry Pi.
  – Not installing the EVA 3.0 print head.
  – Modifying the CoreXY idler assemblies with slimmer profile components. Replace the *xy_idler_left* and *xy_idler_right* printed parts with *xy_idler_upper_new* included in the file repository. Replace the two *idler_plate* metal parts with *xy_idler_lower_new* printed parts included in the file repository.
  – Lower the top horizontal aluminum extrusion on the two Z-motor side 160 mm from the top (Fig. 7).
• Insert a 390 Ω resistor in thermistor pin J46 of the BTT Octopus mainboard.
• Use four *RatRigSpacerPlate*, sixteen M6 15 mm screws, and sixteen M6 T-slot fasteners to connect the two printers together. Connect the four spacer plates to the vertical aluminum extrusion on the shared printer side with two Z-motors.

**Fig. 5:**
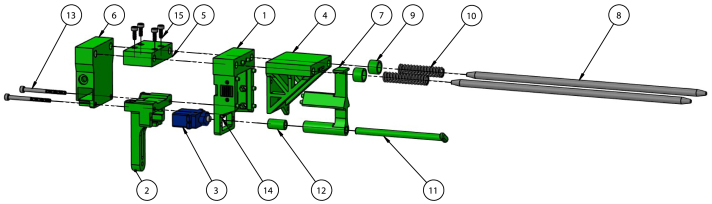
Exploded tool mount assembly. (1) *Rail Mount - Front*, (2) *Rail Mount - BLT Mount*, (3) SG90 - Micro Servo 9 g - Tower Pro.1, (4) *Rail Mount - Front Support Plate*, (5) *Rail Mount - Connector*, (6) *Rail Mount - Back*, (7) *Rail Mount - Servo Rod Support*, (8) *Tool Mount Rod*, (9) *Rail Mount - Spring Holder*, (10) Compression spring [20], (11) *Servo Lock Key*, (12) *Servo Gear Shaft Coupling*, (13) M3 Nut [20], (14) M3 × 8 mm Screw [20].

**Fig. 6:**
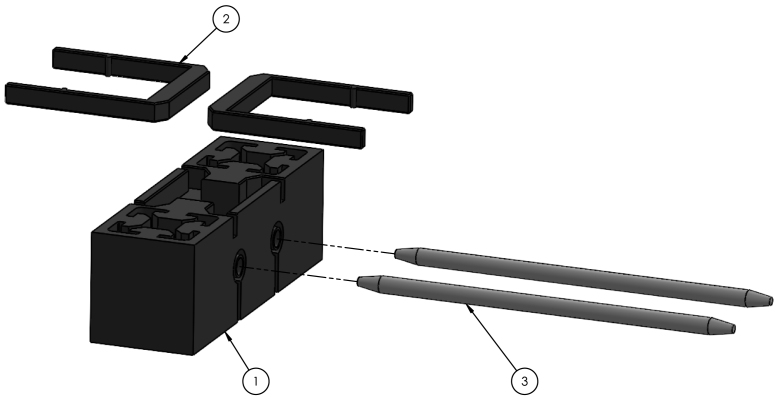
Tool dock exploded view. (1) *Double Sleeve Mount - Upper*, (2) *Double Sleeve Mount - Snap Connector*, (3) *Tool Dock Rod*.

**Fig. 7:**
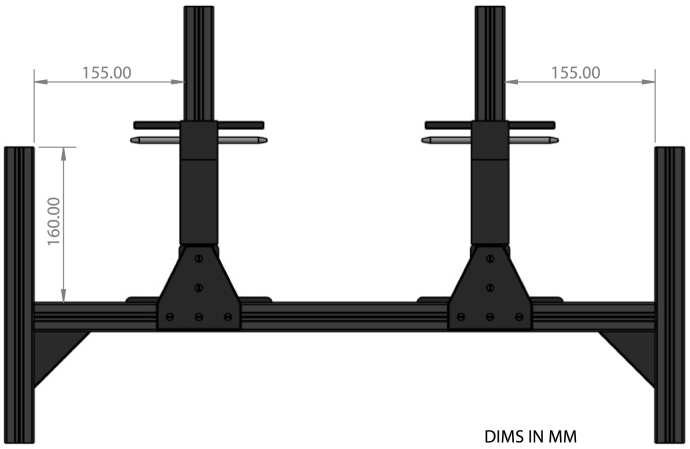
Tool dock layout showing location of tool dock towers. Includes COTS extrusion and brackets [20].

### 5.2 Tool mount assembly

• Starting with the assembled linear rail on the gantry, attach *Rail Mount – Connector* with four M3 × 8 mm screws. Insert the *Tool Mount Rods* so the tapered end points towards the tool dock and sticks out 134 mm from *Rail Mount – Connector*. If difficulty is encountered when inserting the aligning rods, use a drill to slowly spin the rod when inserting.
• Attach *Rail Mount – Front* and *Rail Mount – Back* to the aligning rods. Connect the timing belts with the provided hardware as described in the Rat Rig instruction manual.
• Insert servo motor into *Rail Mount – BLT Mount*. Insert assembly below gantry and secure to *Rail Mount – Front* and *Rail Mount – Back* with two M3 × 40 mm screws and two M3 nuts.
• Connect servo motor to pin J74 on BTT Octopus mainboard using 22AWG wire with Dupont and JST connectors.
• Attach BL Touch mounting hardware (*BLtouch_arm* and *BLtouch_bracket*) to *Rail Mount – BLT Mount* using three M3 × 12 mm screws and three M3 nuts. Attach BL Touch using two M3 × 10 mm screws and two M3 nuts.
• Connect BL Touch to pin J43 on BTT Octopus mainboard using 22AWG wire with Dupont and JST connectors.
• Attach *Rail Mount – Front Support Plate* to *Rail Mount – Front*.
• Insert *Servo Lock Key* into *Rail Mount – Servo Rod Support* and attach *Servo Gear Shaft Coupling* with super glue.
• Set servo position to 25 using G-code M280 P1 S25. Attach assembly with *Servo Lock Key* in horizontal orientation to *Rail Mount – Front Support Plate*. A wooden toothpick can be optionally inserted through the holes in *Rail Mount – Front Support Plate* and *Rail Mount – Servo Rod Support* to better secure the servo rod while still being able to breakaway.
• Ensure the *Tool Mount Rods* protrude 70 mm out of *Rail Mount – Front Support Plate*.
• Insert a 1.6 mm thick washer onto each tool mount aligning rod.
• Attach spring to *Rail Mount – Spring Holder*. Insert assembly onto tool mount aligning rod, spring holder side first.
• An exploded model view of the tool mount is shown in Fig. 5.

### 5.3 Tool dock assembly

• Ensure the top horizontal aluminum extrusion on the two Z-motor side is lowered 160 mm from the top.
• Cut to length and attach two 250 mm long aluminum extrusion, one on each printer side, vertically using extended corner brackets, tee brackets, M6 × 15 mm screws, and T-slot fasteners. Ensure both vertical extrusions are aligned with each other and are positioned 155 mm from either end as shown in Fig. 7.
• Slide 86 mm worth of *Double Sleeve Mount – Spacer* onto each vertical aluminum extrusion.
• Press fit two *Double Sleeve Mount – Snap Connector* onto *Double Sleeve Mount Upper* ensuring the snap connectors rest in a neutral position.
• Insert two *Tool Dock Rods* into the middle of *Double Sleeve Mount Upper*. If difficulty is encountered when inserting the aligning rods, use a drill to slowly spin the rod when inserting.
• Slide *Double Sleeve Mount Upper* onto vertical aluminum extrusion.
• Use a tool holder to check vertical alignment between the dock and tool mount. To adjust vertical alignment, change the height of the *Double Sleeve Mount – Spacers*.
• An exploded model view of the tool dock is shown in Fig. 6.

### 5.4 Tool holder assembly

**Fig. 8:**
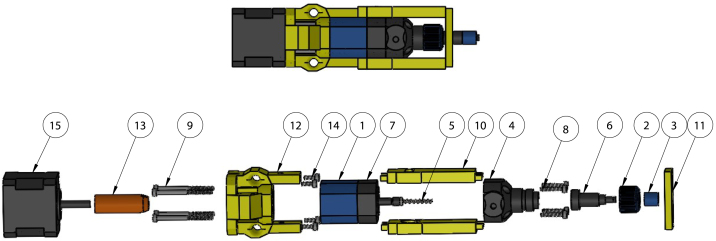
Assembled (top) and exploded (bottom) tool holder with eco-PEN, rotor adapter, stepper motor, and screws. (1) eco-PEN300 Rotor Base, (2) eco-PEN300 Cap Nut, (3) eco-PEN300 Tip Adapter, (4) eco-PEN300 Ink Housing, (5) eco-PEN300 Auger, (6) eco-PEN300 Stator, (7) eco-PEN300 Cavity Seal, (8) M3 × 16 mm Screw [20], (9) M3 × 40 mm Screw [20], (10) *Tool Housing - Key Slot*, (11) *Tool Housing - Nozzle Clip*, (12) *Tool Housing - Stepper Motor Mount Adapter*, (13) *Rotor Adapter*, (14) M3 × 8 mm Screw [20], (15) NEMA 17 Stepper Motor.

**Fig. 9:**
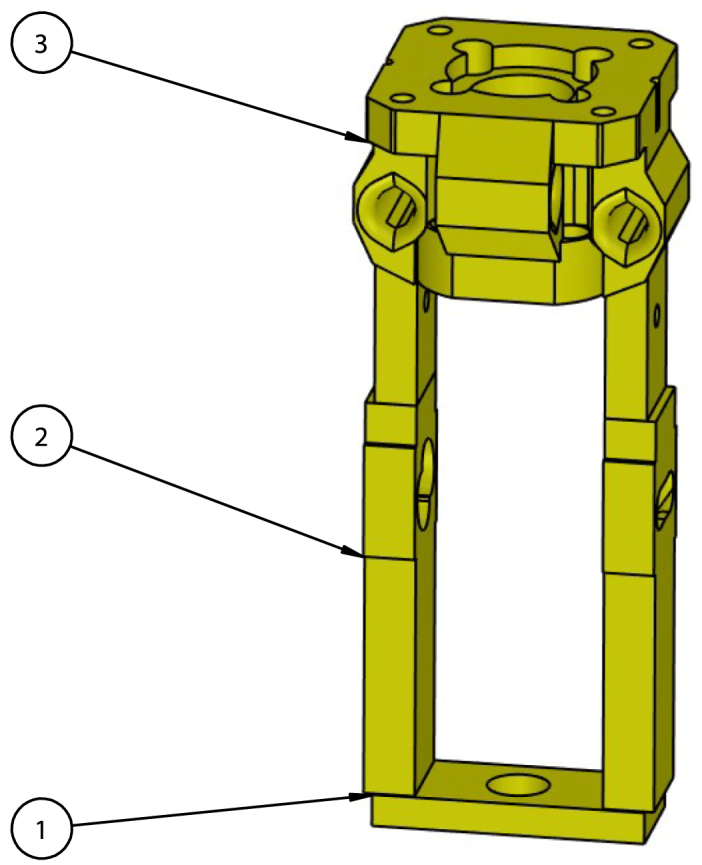
Assembled tool holder. (1) *Tool Housing - Nozzle Clip*, (2) *Tool Housing - Key Slot*, (3) *Tool Housing - Stepper Motor Mount Adapter*.

• Remove the stock servo motor from the eco-PEN300.
• Attach the *Tool Housing – Key Slot*, *Tool Housing – Nozzle Clip*, and *Tool Housing – Stepper Motor Mount Adapter* to the eco-PEN.
• Attach the *Rotor Adapter* to the NEMA 17 stepper motor.
• Use four M3 × 10 mm screws to attach the *Tool Housing – Stepper Motor Mount Adapter* to the stepper motor.
• An exploded model view of the tool holder and extruder assembly is shown in Fig. 8. An assembled tool holder without an extruder is shown in Fig. 9.

### 5.5 Firmware flashing

• Unzip the *Marlin Firmwar_x.zip* file included with this paper and open with Visual Studio Code. Follow prompts to install the Auto Build Marlin extension and build the firmware. Detailed instructions on how to build Marlin in Visual Studio Code can be found at https://marlinfw.org/docs/basics/install_platformio_vscode.html.
• Navigate to *.pio* → *build* → *STM32F446ZE_btt* → *firmware.bin*.
• Save the *firmware.bin* file to a microSD card.
• With the printer turned off, insert the microSD card into the microSD slot on the BTT mainboard.
• Turn the printer on. The printer is now flashed and the microSD can be removed. If successful, the *firmware.bin* file on the microSD will be replaced with *FIRMWARE.CUR*.

### 5.6 Arduino setup and wiring

**Fig. 10:**
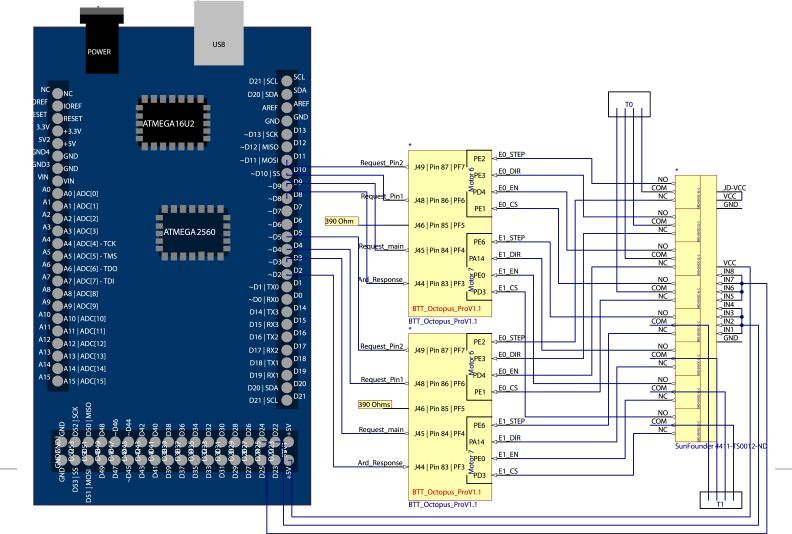
Pin diagram showing wiring between Arduino Mega, BTT Octopus mainboard, SunFounder relay, and extruder stepper motors (T0, T1).

• Connect the four GPIO pins from the Octopus Pro v1.1 main board to the four corresponding GPIO pins on the Arduino, for both printers (Fig. 10).
• Connect the motor ports of the right printer to the normally closed pins on the SunFounder relay. Connect the left printer to the normally open pins on the relay. Connect the extruder motors to the common pins on the relay. Connect the input triggers, 5 V voltage supply, jumper, and ground as shown in Fig. 10.
• Load *ExtruderInterface.ino* onto the Arduino, confirming the pin configuration matches the current physical setup. The Arduino should have one pin configured for each extruder and four pins configured for each printer, one for the tool change request, two for tool change requests, and one for tool change approval.

**Fig. 11:**
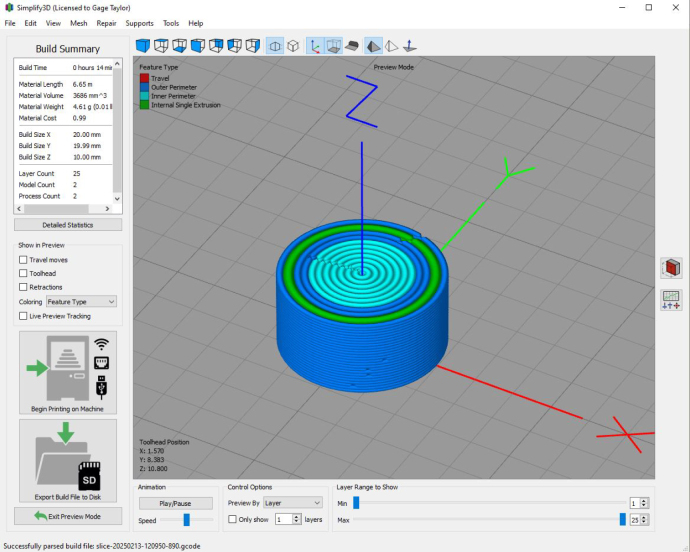
Sliced concentric cylinders in Simplify3D. Factory file available in repository associated with this paper.

## 6 Operation instructions

### 6.1 Extruder preparation

• Load ink into 30 cc syringe barrels, making sure to remove any trapped air pockets. Insert a piston behind the ink.
  – Ink used for concept validation of this hardware was made from cake frosting with added sugar granules, degassed in a FlackTek SpeedMixer DAC 330-100 Pro.
• Connect the loaded ink cartridges to the eco-PEN using 1/8 NPT extender and 90 degree adapters.
• Attach air supply adapters to the ink cartridges, supplied with 30 psi of compressed air.
• Attach a dispensing nozzle to end of eco-PEN.
• Prime the eco-PEN using G-code “T0”, “G1 E100 F100”, where T specifies the tool number, E specifies mm of extrusion, and F specifies speed. Continue to extrude by increasing the E value until a steady flow of material exits the nozzle.

### 6.2 Printer operation

• For multi-material parts, create separate STL files for each different material, aligned to the same reference frame. For example, this can be achieved in a SolidWorks part file by unchecking “Merge Result” while building a part, resulting in separate bodies. When exporting the STL files, turn off the visibilities for all but one body at a time.
• Open the Simplify3D factory file *S3D.factory* included with this article. Remove example parts.
• Load STL files into Simplify3D. Go to “Edit” and click “Align Selected Model Origins”.
• Under “Processes”, double click an extruder and then click “Select Models”. Select which model is associated with that extruder.
• Adjust material properties for each extruder as desired.
• When ready to slice, click “Prepare to Print”. Save the file to your device (Fig. 11).
• Run the post processor script to insert tool changing G-code. Follow prompts to specify left or right printer.
• Turn on the printers and connect to a computer with a USB cable.
• On Pronterface connect to the printer by setting the Baudrate to 250 000, selecting the applicable com port, and clicking “Connect”. The printer’s com port can be found in the Device Manager app if using Windows.
• Home the X, Y, and Z directions.
• Move the end effector to the desired print location, move down to Z-height level 0.
• Set initial pin states for both printers with the following lines of G-code: “M42 P84 T1”; “M42 P86 T1”; “M42 P87 T1”.
• Press the reset button on the Aurduino.
• Click “Load File” to select the executable modified G-code file.
• Ensure ink cartridges are loaded and extruders are primed.
• Click “Run” to start the print.

### 6.3 Extruder cleaning

• Remove the tool holder from the extruder and disassemble the eco-PEN. Thoroughly clean all ink contacted components.
• Reuse or properly dispose of ink cartridges, adapter fittings, dispensing nozzles, and leftover ink as applicable.

**Fig. 12:**
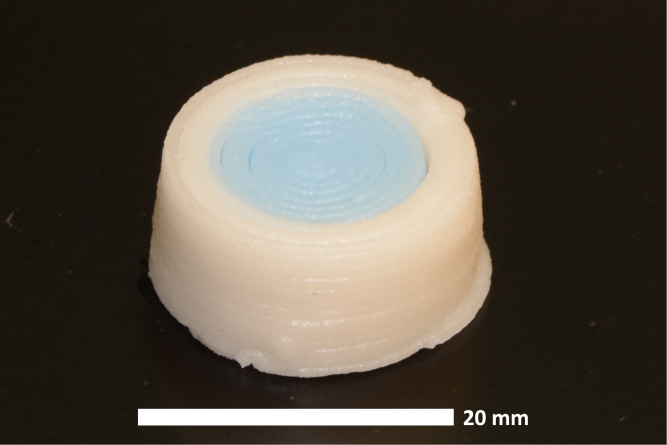
Multi-material test print with equal amounts of blue and white ink printed using the dual Rat Rig. (For interpretation of the references to color in this figure legend, the reader is referred to the web version of this article.)

**Fig. 13:**
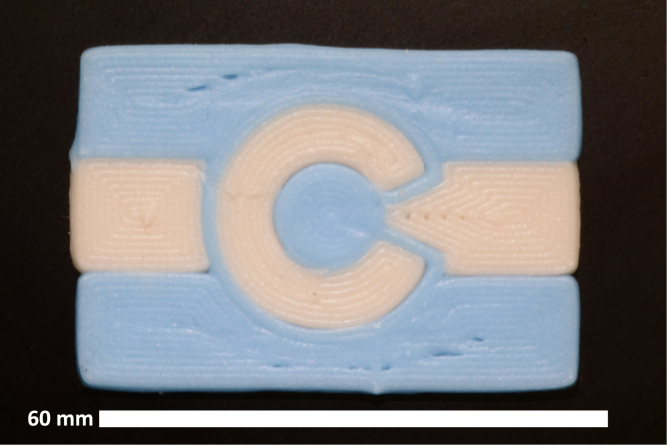
Multi-material test print of the Colorado flag.

## 7 Validation and characterization

The tool changing and sharing system was validated with test prints and repetitive tool change cycle tests. Initial tool change failures were attributed to snagging wires which obstructed tool holder movement. Adding weights to the ends of the wires and airlines facilitated retraction, preventing tool change failures. Further improvements can be achieved by implementing drag chains or similar cable management solutions. After addressing cable management, two tests were conducted to assess the reliability of the tool changer. One test involved 100 cycles of a single printer picking up a tool, tracing a circle on the build plate, dropping off the tool, and switching to a new tool. The printer successfully completed 100 tool change cycles without failure. The second test was similar, but involved both printers operating simultaneously and coordinating tool pick ups and drop offs. Both of the printers successfully completed 100 tool changing and sharing cycles without failure.

To further validate the hardware, colored cake frosting with added sugar granules was used to demonstrate printing with solids loaded inks. Since ViscoTec progressive cavity pump extruders enable printing with a wide range of ink rheological properties, this easy to manufacture, low cost, and non-hazardous ink was selected to demonstrate the efficacy of the system and enable easy replicability. Multi-material concentric cylindrical components were created in SolidWorks and sliced using Simplify3D (factory file available in repository). The compiled G-code was then modified using the post-processor before being run on the printers. Frosting test prints demonstrate the multi-material print capabilities of the tool changing and sharing system and are shown in Fig. 12, Fig. 13. Both printers were able to successfully operate independently while coordinating tool sharing. When a printer needed to exchange a tool, it first returned any tool it was holding, then paused and signaled the Arduino, specifying the desired tool. Once available, the Arduino would notify the printer, and then resume printing. This request and wait system worked well for multi-material components with similar amounts of each material per layer. However, for prints dominated by a single material, while tool sharing functioned properly, the extended pauses between tool changes were suboptimal. Implementing better optimized tool sharing strategies could improve efficiency in such cases. Repeated tool change cycle tests demonstrated the hardware’s reliability throughout the course of a print, with both printers successfully coordinating tool sharing while operating simultaneously.

Tool changing and tool sharing can improve the efficiency of printing operations. Tool changing enables a single printer to utilize multiple extruders without greatly reducing the printer’s build volume. The simplest multi-extruder configuration mounts multiple extruders directly onto a printer’s gantry. However, as the number of extruders increases, the effective build area decreases as the gantry needs to translate back and forth further to reach the mutually accessible print area. Tool changing prevents this limitation, allowing multiple extruders to be used without sacrificing build space, making it ideal for creating multi-material components.

Tool sharing can further improve efficiency by increasing extruder utilization. In single material parts, additional extruders can be mounted on the printer’s gantry to increase throughput. However, single material parts are often better suited for conventional manufacturing techniques. In contrast, multi-material components with complex geometries can benefit from additive manufacturing. Traditional multi-material printing methods only use one extruder at a time, leading to low equipment utilization as inactive extruders sit idle. Tool sharing increases extruder duty cycles by sharing tooling between multiple printers. This increases printing throughput while reducing extruder capital investment, which in the case of this printer setup is the most expensive component.

To assess the efficiency of the tool changing system, several timed tests were conducted for a concentric cylindrical part comprised of two materials of equal volume. For two printers tool changing and tool sharing, it took 26 min and 55 s to print two parts, one for each printer. For a single printer only tool changing, it took 23 min and 20 s for one part since there was no downtime waiting for tools to become available. To simulate a single printer with two 30 mm wide eco-PEN300 extruders fixed on the gantry 40 mm apart, G-code was inserted to cause the printer to translate 40 mm when switching between different materials. This took 18 min and 5 s for one part. Since the tool changing and sharing system takes 26 min and 55 s for two parts, the throughput is on average one part every 13 min 27 s. Tool changing and sharing reduced print time by 4 min 38 s, a 26% reduction. While the theoretical maximum reduction is 50%, this is difficult to achieve due to extruder down time during tool changing, which is not experienced with side-by-side fixed gantry mounted extruders.

In addition to lowering print time, tool sharing reduces extruder cleaning and maintenance operations. DIW 3D printing can be a labor-intensive process as extruders need to be commissioned and decommissioned between periods of use. While commissioning is often straightforward and involves priming the extruders, decommissioning and cleaning are time-consuming. For inks which are hazardous, require special disposal, or involve cleaning solvents, minimizing operator exposure and hazardous waste generation is imperative. By reducing the total number of extruders, tool sharing decreases cleaning, maintenance, and disposal operations.

This tool changing and tool sharing hardware enhances DIW printing by improving multi-material printing capabilities, increasing production efficiency, lowering overall system costs, and reducing operator workload. This work can be expanded upon in the future by developing a more sophisticated tool sharing algorithm, integrating automated cleaning processes, adding closed-loop extrusion feedback control, and enabling compatibility with additional printer designs.

## CRediT authorship contribution statement

**James P. Verheyden:** Writing – review & editing, Writing – original draft, Visualization, Validation, Software, Resources, Methodology, Investigation, Conceptualization. **Bryce Huffaker:** Writing – review & editing, Writing – original draft, Visualization, Software, Methodology, Investigation, Conceptualization. **Max J. Sevcik:** Writing – review & editing, Visualization, Methodology, Conceptualization. **Isaac Snyder:** Visualization, Software, Methodology, Conceptualization. **Finnegan Wilson:** Writing – review & editing, Conceptualization. **Grace I. Rabinowitz:** Writing – review & editing, Conceptualization. **Carter Watkins:** Methodology. **Elbert Caravaca:** Supervision, Funding acquisition. **Edward G. Tersine:** Supervision, Funding acquisition. **Veronica Eliasson:** Writing – review & editing, Supervision, Project administration, Funding acquisition, Conceptualization.

## Declaration of competing interest

The authors declare that they have no known competing financial interests or personal relationships that could have appeared to influence the work reported in this paper.
